# Editorial: Recent developments in pancreatic cancer radiotherapy, vol II

**DOI:** 10.3389/fonc.2024.1541855

**Published:** 2024-12-19

**Authors:** Antonio Pontoriero, Gianluca Ferini

**Affiliations:** ^1^ Radiation Oncology Unit, Department of Biomedical, Dental and Morphological and Functional Imaging Sciences, University of Messina, Messina, Italy; ^2^ Istituto Oncologico del Mediterraneo, Viagrande, Italy; ^3^ Department of Medicine and Surgery, University Kore of Enna, Enna, Italy

**Keywords:** pancreatic cancer, radiotherapy, SBRT, MR-linac, locally advanced, neoadjuvant chemotherapy, overall survival, diffusion-weighted MRI

This Research Topic examines recent advances in the treatment of patients with locally advanced and metastatic pancreatic cancer, highlighting innovative approaches in both therapy and prognosis assessment. The role of radiation therapy (RT) in managing pancreatic cancer, particularly in locally advanced and metastatic stages, remains an area of active investigation. While RT can enhance local control and provide symptom relief, its potential to translate these effects into long-term survival benefits is still debated. The integration of RT with chemotherapy has shown encouraging results, though the degree of benefit varies significantly among patients. Yang et al. contributed to this discussion by addressing key topics of interest in pancreatic carcinoma: the role of Stereotactic Body Radiotherapy (SBRT) and Neoadjuvant Therapy (NAT), as well as the limitations and challenges associated with Immunotherapy (IT).

Stereotactic Body Radiotherapy (SBRT) requires further investigation to validate its efficacy as a neoadjuvant therapy for pancreatic cancer. Nevertheless, SBRT offers distinct advantages in this setting, including shorter treatment duration, a reduced risk of chemotherapy interruption, and the delivery of a higher biologically effective dose (BED), which may enhance local tumor control and potentially translate into survival benefits. Specifically, a BED exceeding 70 Gy has been associated with improved survival in pancreatic cancer (1).

Neoadjuvant therapy (NAT) followed by surgical resection has demonstrated several advantages over immediate resection. These include enabling more patients to receive systemic therapy, increasing the rate of R0 resections, and improving overall survival. Moreover, NAT can downstage tumors, allowing initially inoperable cases to become suitable for resection, thereby offering the potential for curative surgery and better survival outcomes (2).

Despite its promise, immunotherapy (IT) in pancreatic cancer faces significant challenges. The low mutational burden of pancreatic tumors limits the activation of an effective immune response, while the immunosuppressive tumor microenvironment further reduces IT efficacy (3). Consequently, IT alone has demonstrated limited effectiveness in this context. However, combining IT with radiation therapy (RT) could represent a promising strategy to enhance the therapeutic response by modulating the tumor microenvironment and potentiating the immune response (4, 5).

For patients with non-metastatic pancreatic cancer (cT1-4N0-1M0), Yang et al. analyzed the efficacy of neoadjuvant chemotherapy (NACT) versus neoadjuvant chemotherapy and radiotherapy (NARCT) before surgery, using data from the SEER database, which included 723 patients. The results showed that the median overall survival (OS) was 30 months for NACT compared to 25 months for NARCT, while the median cancer-specific survival (CSS) was 33 months versus 26 months, respectively. Age, lymph node positivity, and the use of NARCT were identified as adverse prognostic factors. These findings suggest that NACT may be the optimal neoadjuvant treatment option for patients with non-metastatic pancreatic cancer.

To evaluate the benefits of radiation therapy (RT), Cao et al. analyzed patients with unresectable locally advanced pancreatic cancer (LAUPC) and metastatic pancreatic cancer (MPC), comparing the outcomes of chemotherapy (CT) alone with combination therapy (CMT) that includes RT. Through a retrospective analysis of patients treated at a single institution and data from the SEER database, the results demonstrated that CMT significantly improved overall survival (OS) compared to CT alone. In the institutional cohort, CMT showed a median OS of 22.2 months compared to 11.8 months for CT. These results were also validated in the SEER database, suggesting that integrating RT with CT could be an effective strategy for the treatment of LAUPC and MPC. The study identified several key predictors of survival, including treatment group (CMT vs. CT), primary tumor site, T stage, N stage, AJCC stage, and the presence of liver metastases.

The apparent divergence in the usefulness of RT in non-metastatic stages between the results of Yang et al. and Cao et al. could stem from the different methods used by the two research teams to query the SEER database, covering slightly different time periods (2010–2017 vs. 2010–2020). While Yang et al. included all non-metastatic patients classified according to the TNM staging system, Cao et al., apart from metastatic patients, focused exclusively on non-metastatic patients defined as having LAUC based on a surgical criterion of resectability. This specific subgroup of non-metastatic T4 patients may have been underrepresented or inconsistently grouped with lower T stages in Yang et al.‘s cohort, potentially obscuring the benefits of RT in this particular patient setting. Considering all these factors, the results do not appear contradictory and RT may be useless only in resectable tumors. Furthermore, the findings by Cao et al. strongly support the need for further trials investigating radiation dose escalation, as preliminarily explored by Parisi et al., with promising outcomes (2).

Ferris et al. presented an innovative approach to SBRT for pancreatic cancer using a magnetic resonance imaging-guided linear accelerator (MR-linac). The study involved 26 patients and demonstrated that abdominal compression reduced tumor motion, thereby improving treatment precision. This technique increased the duty cycle compared to gating and breath-hold methods, making the treatment more accessible. By reducing tumor motion, abdominal compression allowed for greater treatment accuracy, smaller margins, and an increased prescribed dose. As a result, the approach improved the mean prescription isodose coverage at the target volume (77.9%) and minimized interference with nearby at-risk structures. The clinical results showed favorable local control, with 80.8% of patients remaining free of local progression, and a median of 5.6 months to local progression (19%). Additionally, 53.8% of patients experienced metastatic progression, with a median of 3.1 months to metastatic progression. The therapy was generally well tolerated, although some patients experienced toxicities of grade 3 or higher.

To predict overall survival, Bisgaard et al., in a study of 45 patients with locally advanced pancreatic cancer treated with SBRT, proposed the use of diffusion magnetic resonance imaging (DWI). The results suggest that longitudinal DWI parameters have the potential to personalize treatment and serve as prognostic biomarkers. The derived DWI parameters used for predicting overall survival include the apparent diffusion coefficient (ADC) and parameters obtained through a decomposition method. The analysis of DWI data was performed using monotonous slope non-negative matrix factorization (msNMF). The best multivariate model incorporated two DWI parameters based on decomposition, considering both baseline values and changes during treatment. The overall survival (OS) prediction results indicated that the median OS from the first day of SBRT was 15.5 months (95% CI: 13.2-20.6).

Further studies are needed to confirm and determine the optimal neoadjuvant therapy modality for pancreatic cancer in order to improve prognosis. However, we hope that the results presented in this Research Topic will be useful to our colleagues, clinicians, and researchers in understanding the role of radiotherapy in the treatment of pancreatic cancer, particularly in locally advanced and metastatic cases, which remain under investigation. We would like to thank and congratulate all the authors who contributed their work to this Research Topic.
